# Familial Bainbridge‐Ropers syndrome: Report of familial *ASXL3* inheritance and a milder phenotype

**DOI:** 10.1002/ajmg.a.62981

**Published:** 2022-09-29

**Authors:** Schaida Schirwani, Emily Woods, David A. Koolen, Charlotte W. Ockeloen, Sally Ann Lynch, Karl Kavanagh, John M. Graham, Katheryn Grand, Tyler Mark Pierson, Jeffrey M. Chung, Meena Balasubramanian

**Affiliations:** ^1^ Department of Oncology & Metabolism University of Sheffield Sheffield UK; ^2^ Sheffield Clinical Genetics Service Sheffield Children's NHS Foundation Trust Sheffield UK; ^3^ Department of Human Genetics Donders Institute for Brain, Cognition and Behavior, Radboud University Medical Center Nijmegen Netherlands; ^4^ Department of Clinical Genetics Children's Health Ireland Dublin Ireland; ^5^ Division of Medical Genetics, Department of Pediatrics Cedars‐Sinai Medical Center Los Angeles California USA; ^6^ Department of Pediatrics Cedars‐Sinai Medical Center Los Angeles California USA; ^7^ Department of Neurology Cedars‐Sinai Medical Center Los Angeles California USA; ^8^ Cedars‐Sinai Center for the Undiagnosed Patient Cedars‐Sinai Medical Center Los Angeles California USA; ^9^ Board of Governors Regenerative Medicine Institute Cedars‐Sinai Medical Center Los Angeles California USA

**Keywords:** *ASXL3*, Bainbridge‐Ropers syndrome, developmental delay

## Abstract

De novo truncating and splicing pathogenic variants in the Additional Sex Combs‐Like 3 (*ASXL3*) gene are known to cause neurodevelopmental delay, intellectual disability, behavioral difficulties, hypotonia, feeding problems and characteristic facial features. We previously reported 45 patients with *ASXL3*‐related disorder including three individuals with a familial variant. Here we report the detailed clinical and molecular characteristics of these three families with inherited *ASXL3*‐related disorder. First, a father and son with c.2791_2792del p.Gln931fs pathogenic variant. The second, a mother, daughter and son with c.4534C > T, p.Gln1512Ter pathogenic variant. The third, a mother and her daughter with c.4441dup, p.Leu1481fs maternally inherited pathogenic variant. This report demonstrates intrafamilial phenotypic heterogeneity and confirms heritability of *ASXL3*‐related disorder.

## 1 INTRODUCTION

*ASXL3*‐related disorder, also known as Bainbridge‐Ropers syndrome (BRPS), is a rare multisystemic disorder caused by truncating and splicing pathogenic variants in the additional sex combs‐like 3 (*ASXL3*) gene resulting in loss‐of‐function as the predominant disease mechanism. It is characterized by neurodevelopmental delay, moderate to severe learning difficulties, behavioral problems, hypotonia and feeding problems. Characteristic craniofacial features include prominent forehead, down‐slanting palpebral fissures, prominent nasal bridge, broad nasal tip, low columella, high‐arched palate, and crowded teeth.

Here, we report detailed clinical and molecular characteristics of three families with *ASXL3*‐related disorder which represents the first detailed description of familial *ASXL3*‐related disorder. Almost all patients with *ASXL3*‐related disorder have de novo pathogenic variants in *ASXL3* (Balasubramanian et al., 2017; Bainbridge et al., 2013; Dinwiddie et al., 2013; Schirwani et al., 2021). No clear genotype–phenotype correlation exists and there is significant phenotypic heterogeneity between individuals. Intrafamilial phenotypic variation has not been previously explored and there has not been a prior detailed report of familial cases.

## 2 CLINICAL DESCRIPTION

### 2.1 Family 1

The proband was referred for genetic evaluation at 4 years of age because of developmental delay and possible episodes of apnoea. Whole exome sequencing found an *ASXL3*; Chr18(GRCh37):g.31320159_31320160del; NM_030632.1:c.2791_2792del, p.Gln931fs variant. This was confirmed to be paternally inherited following specific testing of *ASXL3* variant in the parents.

This patient was born at term via normal delivery weighing 3.85 kg (75th centile). At age 4 years, his height was 98.5 cm (20th centile), weight was 13.6 kg (5th centile) and head circumference of 47.5 cm (1st centile). He had initial feeding difficulties with poor sucking reflex in the neonatal period. His fontanelles closed prematurely.

Developmentally, motor milestones were achieved appropriately but speech and language development were severely delayed. At 4 years of age, he had language skills in keeping with a 12‐month‐old and had learning difficulties with an IQ of 65. He had frequent temper tantrums but no additional features of autism or attention deficit hyperactivity disorder (ADHD). He received eye movement desensitization and reprocessing (EMDR) therapy because of severe anxieties, including a fear of vomiting or having diarrhea.

He was not toilet‐trained until 5 years of age and continued to have problems with eating. He attended a special day care nursery. As an infant, he had multiple upper respiratory tract infections. He was also investigated for possible episodes of sleep apnoea. Polysomnography showed few central apnoeas during rapid eye movement sleep cycles. Hering‐Breuer reflex showed no evidence of pathological breathing. He subsequently had an adenotonsillectomy with grommet insertion at the age of 3 years. Investigation of an asymptomatic supraclavicular swelling revealed a dilated subclavian artery measuring approximately 3 cm (maximum dilatation 15 mm). In the absence of clinical signs of a connective tissue disorder, a WES gene panel for thoracic aortic aneurysm and dissection was carried out and was negative. No other aneurysms were seen on full body magnetic resonance angiography (MRA).

Currently, he attends regular primary education with extra support and receives additional home support.

His father was born at term, with a birth weight of 3.6 kg. Motor and speech and language milestones were met appropriately. Growth parameters for the proband's 37‐year‐old father were: height 182 cm (80th centile) and weight 90 kg (90th centile).

He was diagnosed with ADHD at 11 years and had gone to a special school during childhood. He had difficulties with reading and information processing.

A formal neuropsychological evaluation was performed at the age of 37. The Wechsler adult intelligence scale (WAIS‐IV‐NL) showed a total IQ of 69 with deficiencies in performance subtests, working memory and information processing, and a verbal IQ of 91 (disharmonic profile). He had a part‐time job as a courier.

**FIGURE 1:**
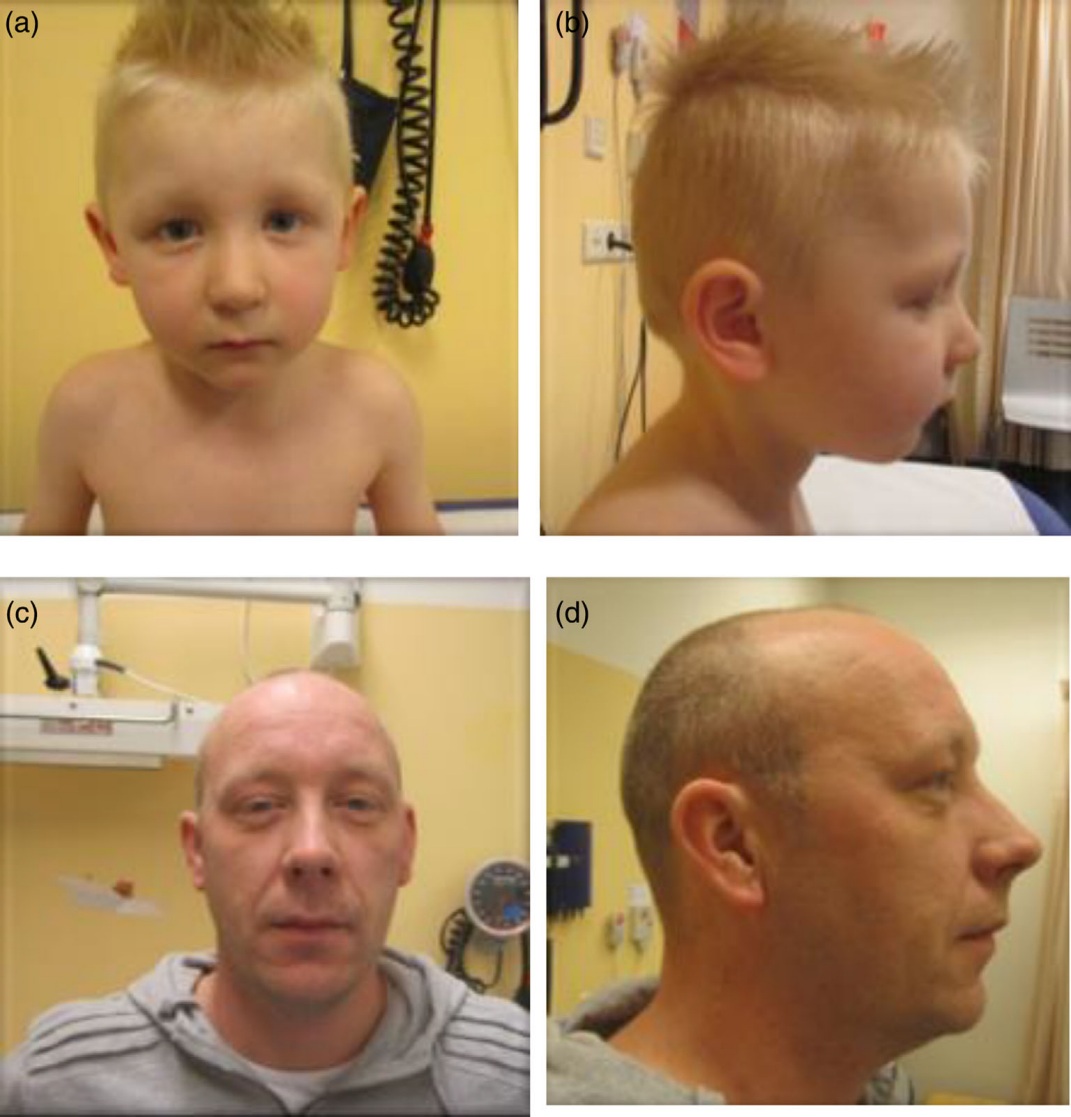
(a, b) Proband, family 1. Features including down‐slanting palpebral fissures, prominent columella, small mouth, mild micrognathia. (c, d) Father of proband, family 1. Features including down‐slanting palpebral fissures, prominent columella, small mouth, mild micrognathia

Dysmorphic features that were present in both the proband and his father included: down‐slanting palpebral fissures, prominent columella, small mouth, high arched, narrow palate and mild micrognathia (Figure 1a,b and Figure 1c,d). There were no additional features of Marfanoid habitus in either individual. Teeth abnormalities included small teeth in the proband, and overcrowded teeth in the father.

### 2.2 Family 2

This family of mother, daughter and son, were referred for genetic evaluation, initiated by severe global developmental delay in the son at 4 months of age. The family were enrolled into the Deciphering Developmental Delay (DDD) study which identified a c.4534C > T, p.Gln1512Ter pathogenic variant, which occurred *de novo* in the mother, and was maternally inherited by both her children. Testing was done through the Deciphering Developmental Disorders Study at the Wellcome Trust Sanger Institute using Agilent 2×1M for array‐based comparative genomic hybridization (aCGH), Illumina 800 K SNP genotyping to identify copy number variants, and Agilent SureSelect 55 MB Exome Plus with Illumina HiSeq for exome sequencing (Wright et al., 2015). Putative de novo mutations were identified from exome data using DeNovoGear software, interpreted for their clinical relevance based on ACMG/AMP and ACGS guidelines and were validated using targeted Sanger sequencing (Richards et al., 2015; Ellard et al., 2020).

The mother was born at 42 weeks gestation by emergency caesarean section due to umbilical prolapse, weighing 3.9 kg (77th centile). Final growth parameters were also all within normal range; height 168 cm (85th centile), weight 91 kg (78th centile). There was no admission to the Neonatal Unit, and there were no feeding difficulties during the neonatal period. Motor milestones were slightly delayed and she first walked at 18 months. Global neurodevelopmental delay became apparent in nursery and she was later diagnosed with mild intellectual disability and attended a special school. A special programme following on from school presented a job in a supermarket, where she worked for 11 years. In terms of behavioral issues, there were severe temper tantrums in childhood and oppositional behavior during teenage years, but no evidence of autism or ADHD. She was diagnosed with epilepsy, having atonic and absence seizures, aged 7 years and was on antiepileptics until the age of 16 years old. Epilepsy recurred after the birth of her first child and she was started on levetiracetam at that time.

**FIGURE 2:**
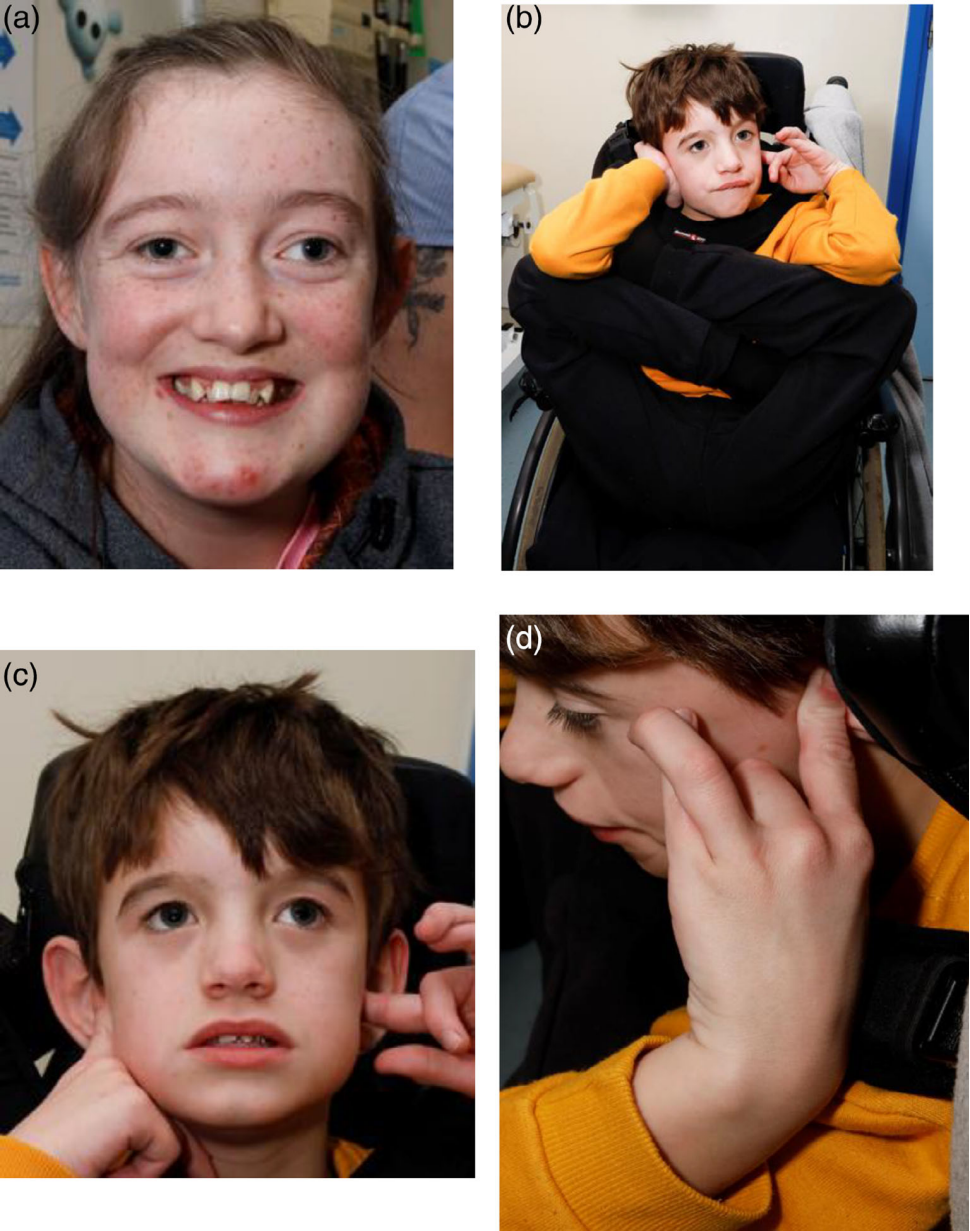
(a) Daughter, family 2, showing tall forehead. (b) Proband, family 2, showing tall forehead. (c) Proband, family 2, showing hypermobility and relative microcephaly. (d) Proband, family 2, showing hypermobility and arachnodactyly

Her daughter was born by elective caesarean section due to maternal epilepsy, weighing 3.3 kg (56th centile). Now at age 15 years, her weight is 52 kg (25th centile), height is 155 cm (9th centile) and she is relatively microcephalic with head circumference of 52 cm (2nd centile) (Figure 2a). During the neonatal period, she was slow to develop effective feeding and had mild failure to thrive. Motor milestones were delayed; she sat at 12 months and walked at 2 years. Speech and language development were also significantly delayed, with her first word at 2.5 years of age. She attended mainstream school with additional support provided for mild intellectual disability and persistent speech and language difficulties. In terms of behavior, she had poor attention, but no stereotypies of autism or ADHD. At 5 years of age, she was diagnosed with epilepsy after having seizures that were characteristically similar to her mother's. Her epilepsy has been well controlled on lamotrigine and oxcarbazepine since. Imaging (MRI) of her head was normal.

Her son was born by emergency caesarean section for breech presentation, weighing 3.9 kg (77th centile). At aged 16 months, growth parameters were: height 75 cm (4th centile), weight 8.6 kg (1st centile) and head circumference 45 cm (1st centile). He was severely hypotonic and had feeding difficulties from birth, with recurrent hypoglycemic episodes, which required percutaneous endoscopic gastrostomy feeding. Developmental milestones were significantly delayed; he first sat independently at 20 months of age and could not walk or talk by 7 years of age. He attended a special school due to severe intellectual disability and significant behavioral difficulties including stereotypies, head rolling and biting. He was extremely hypermobile and repeatedly put his legs behind his ears (Figure 2b–d). Unlike his mother and sister, he had not been diagnosed with epilepsy, but had had one seizure in the past. He had an MRI head as part of investigations into hypotonia, which was normal. Genetic testing also identified two copy number variants (CNV); 2q32.1 (186292094–186,365,446 paternally inherited) and 3p22.2 (37495126–37,595,379 maternally inherited) which are thought not contributory to his phenotype.

The children's father had epilepsy and mild learning difficulties. He could read and write but with some difficulty with concentration and comprehension. He left school at 15 years of age and did not achieve any formal qualifications. There was a history of alcohol abuse since childhood, and depression.

The common dysmorphic feature present in all three individuals was a tall forehead (Figure 2a,b). Despite absence of other dysmorphisms in the mother and daughter, the son additionally had hypermobility, hypotonia, nevus flammeus, long slender fingers and scoliosis (Figure 2b–d). There was evidence of microcephaly in the daughter and son, but normal head circumference in their mother. There is no head circumference measurement for their father for comparison.

### 2.3 Family 3

The proband was referred for genetic evaluation because of intellectual disability, intractable epilepsy and autism. Trio whole exome sequencing identified a heterozygous likely pathogenic frameshift variant that was maternally inherited (c.4441dupC, p.Leu1481fsX12). Previous testing had revealed a *MECP2* variant of unknown significance, which was re‐classified to benign when it was found to be paternally inherited from an asymptomatic male.

This patient was born via caesarean section at 41 weeks due to postmaturity with no complications or need for neonatal admission. There were no feeding difficulties during the neonatal period. Developmental milestones were delayed and she first sat at 15 months, first walked at 2 years, and had developed five words by the age of 2–3 years, after which, speech regressed and she became nonverbal.

At the age of 32 years, growth parameters were: height 158 cm (30th centile), weight 86.8 kg (72nd centile), head circumference 55.4 cm (50th centile). There were no appetite or sleep issues. She is unable to follow commands and remains non‐verbal.

At 12 years of age, she developed epilepsy, and she was initially treated with carbamazepine. However, seizure frequency increased over the subsequent 20 years and she is now on a total of five anti‐epileptic medications (carbamazepine, eslicarbazepine, brivaracetam, perampanel, and clobazam) and has a vagal nerve stimulator inserted. However, she continues to have between 30 and 60 seizures a month despite this. Seizures happened during sleep and wakefulness but much more commonly during sleep. Electroencephalogram (EEG) showed multifocal sharp waves, whilst video EEG showed multifocal seizures; right frontal sharp waves and right frontal paroxysmal fast activity. The paroxysmal fast activity was maximal over the right or left hemisphere accompanied by head deviation in the direction ipsilateral to the more prominent paroxysmal fast activity. Seizure frequency did not seem to be affected by increases in medication. MRI head showed slight prominence of the right temporal horn and slight volume loss of the right hippocampal region without abnormal signaling, but was otherwise normal.

**FIGURE 3:**
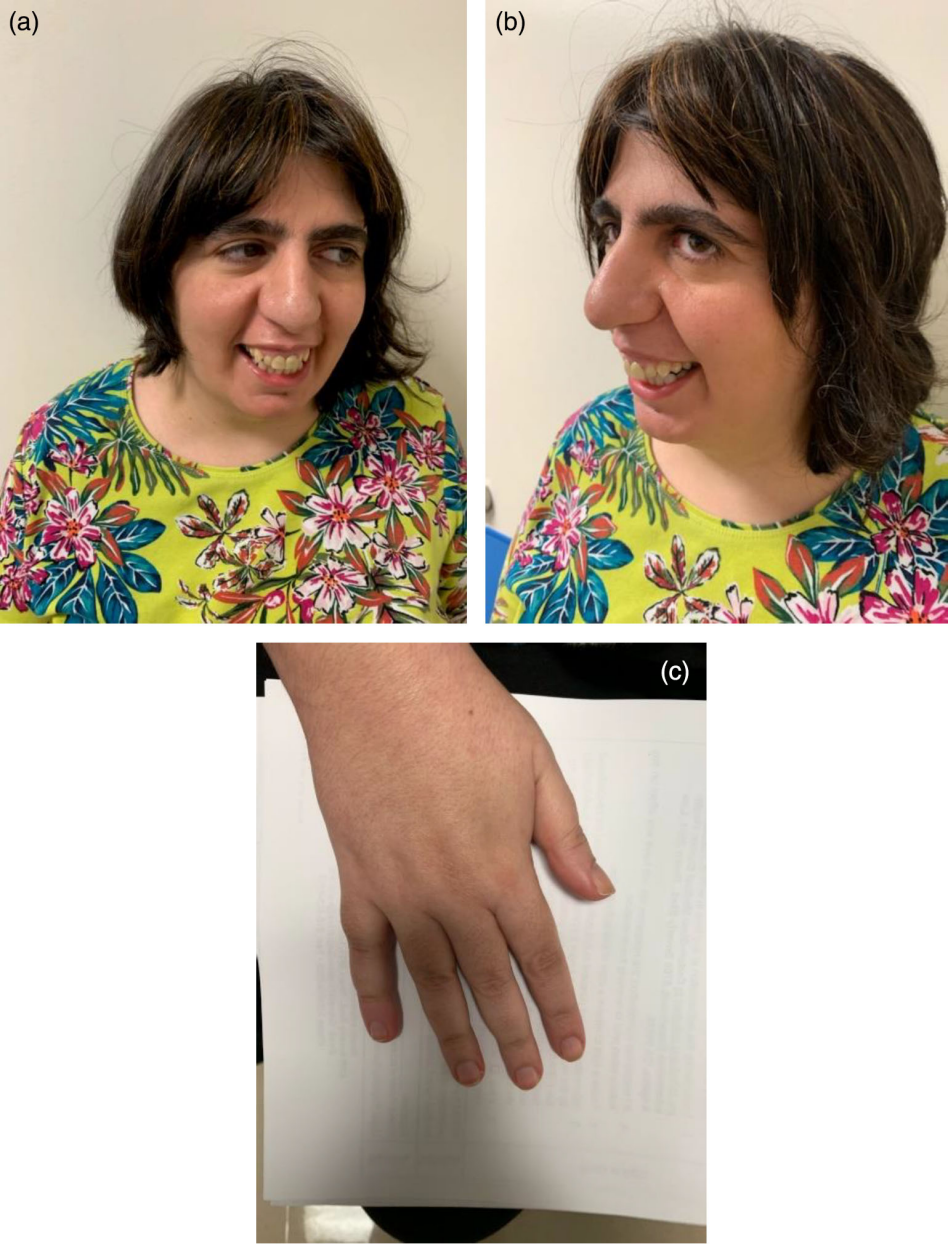
(a) Proband, family 3, showing prominent forehead, down‐slanting palpebral fissures, long eyelashes, full eyebrows, prominent nasal bridge, long, tubular nose, short columella, broad nasal tip and micrognathia. (b) Hands of proband, family 3, showing short first and fifth metacarpals

Facial features included prominent forehead, down‐slanting palpebral fissures, long eyelashes with full eyebrows, prominent nasal bridge, long tubular nose, a short columella and a broad nasal tip (Figure 3a). Her mouth revealed a high‐narrow anterior palate with relative prominence of the maxilla over the mandible and a significant overbite resulting from her relatively small lower jaw (Figure 3a). Her hands tended to be kept in the clenched position at rest, but opened easily with no ulnar deviation. The first and fifth metacarpals appeared somewhat short (Figure 3b) with incomplete transverse palmar flexion creases bilaterally. She walked with a wide‐based, unsteady gait and there was mild kyphoscoliosis. She had irregular breathing and had stereotypies including hand‐flapping. None of these clinical features were present in her mother who has the same variant.

## 3 DISCUSSION

Almost all patients with *ASXL3*‐related disorder have been assumed or confirmed *de novo*. Here we describe three families with inherited *ASXL3*‐related disorder. This has implications for clinical practice. Firstly, the knowledge of possible inheritance from a potentially mildly affected parent may warrant parental testing prior to providing clinical recurrence risk estimates. Secondly, when counseling parents or individuals with *ASXL3*‐related syndrome, it is prudent to inform of the possibility of recurrence and variability of inherited phenotype, so that informed decisions can be made regarding reproductive health of patients. This is especially so with improved access to prenatal exome sequencing for congenital anomalies identified antenatally.

The intrafamilial variability evident in Family 2 and 3 along with previous reports of phenotypic variability in patients with the same *ASXL3* gene variant (Schirwani et al., 2021), underscores the difficulty in establishing clear genotype–phenotype correlation in this disorder (Table 1). In Family 2, the mother and both affected siblings all have significantly different developmental, intellectual and educational profiles; ranging from mild ID in mainstream schooling, to severe ID requiring specialist schooling. In Family 3, the daughter had more significant features of the condition whilst the mother had attended university and had a graduate degree demonstrating the level of intrafamilial variability in inherited *ASXL3*.

**TABLE 1:** Summary of patients

Family	Patient	ASXL3 variant	Inheritance	Dysmorphic features	Clinical features
1	Proband, male child	Chr18(GRCh37):g.31320159_31320160del	Paternally inherited	Down‐slanting palpebral fissures Prominent columella Small mouth High arched Narrow palate Mild micrognathia Small teeth Premature closure of fontanelles Relative microcephaly	Global developmental delay (speech and language and social communication difficulties) Feeding difficulties Temper tantrums Severe anxieties Sleep apnoea Dilated subclavian artery
1	Father of proband	Chr18(GRCh37):g.31320159_31320160del	Unknown	Down‐slanting palpebral fissures Prominent columella Small mouth High arched Narrow palate Mild micrognathia Overcrowded teeth	Intellectual disability (disharmonic profile)
1	Mother of proband	None	N/A	None	None
2	Proband, male child	c.4534C > T (1512 Q/*) p.Gln1512Ter	Maternally inherited	Relative microcephaly Tall forehead Arachnodactyly Scoliosis	Severe global developmental delay across all domains Failure to thrive Hypotonia Feeding problems with hypoglycaemia requiring PEG feeding Intellectual disability Significant behavioral difficulties Hypermobility
2	Mother of proband	c.4534C > T (1512 Q/*) p.Gln1512Ter	De novo	Tall forehead	Global developmental delay Intellectual disability Temper tantrums Epilepsy
2	Sister of proband	c.4534C > T (1512 Q/*) p.Gln1512Ter	Maternally inherited	Relative microcephaly Tall forehead	Global developmental delay (motor, speech and language delay) Failure to thrive Feeding difficulties Poor attention Intellectual disability Epilepsy
2	Father of proband	None	N/A	None	Epilepsy Mild learning difficulties

Family	Patient	ASXL3 variant	Inheritance	Dysmorphic features	Clinical features
3	Proband, female	c.4441dup p.(Leu1481fs)	Maternally inherited	Prominent forehead Down‐slanting palpebral fissures Long eyelashes, full eyebrows Prominent nasal bridge Long, tubular nose Short columella Broad nasal tip High‐narrow anterior palate Prominence of maxilla over mandible with micrognathia (significant overbite) Short first and fifth metacarpals Incomplete palmar crease bilaterally Mild kyphoscoliosis	Global developmental delay Intellectual disability Intractable epilepsy Autism spectrum disorder, nonverbal Hand‐flapping Irregular breathing Unsteady, broad‐based gait
3	Mother of proband	c.4441dup p.(Leu1481fs)	Unknown	None	None (University graduate)
3	Father of proband	None	N/A	None	None

The range of facial dysmorphisms varied across both families, with Family 1 showing more similarity in dysmorphic features, but Families 2 and 3 showing great variability of features. Relative microcephaly was also seen variably in Families 1 and 2. Feeding difficulties were also variable, with the mother of Family 2 and father of Family 1 never having had feeding difficulties, ranging to the son of Family 1 experiencing feeding difficulties in the neonatal period only, to the son of Family 2 requiring PEG insertion for feeding because of poor feeding and failure to thrive. In Family 2, epilepsy was seen in the mother and her daughter, but not in her son, who was otherwise seemingly more severely affected. With families with such phenotypic variability, consideration should be given to potential of somatic mosaicism, however this was excluded in all the families reported here.

There is another patient on Decipher (www.deciphergenomics.org), ID 305643, with the same c.4534C > T, p.Gln1512Ter variant as Family 2, who is reported to have a milder phenotype, which may well explain the milder phenotype in the mother, that may otherwise have gone undetected without parental testing. However, as there was apparent assortative mating with the parents of Family 2, given their intellectual and learning statuses, it is possible that the son inherited an additional so far undetected variant from his father that may have exacerbated his clinical phenotype.

The subclavian artery aneurysm found in the proband in Family 1 is a newly described observation. Although skeletal abnormalities such as Marfanoid habitus, arachnodactyly and scoliosis have been previously described with *ASXL3*‐related disorder, aneurysms have not previously been associated with the condition. Subclavian artery aneurysms are rare, and in children, typically associated with inherited connective tissue disorders such as Marfan syndrome and Loeys Dietz syndrome, infections and vasculitis (Sarkar et al., 1991). They can be asymptomatic but have the potential to cause significant morbidity. The father, who carries the same pathogenic variant, will also be screened for aneurysms with an initial echocardiogram and subsequent evaluation based on this. There have been no prior reports of connective tissue features linked to *ASXL3* in other patients, however, with ASXL3‐related syndrome, we would normally recommend an initial echocardiogram on diagnosis. Further surveillance would be dependent on any abnormal initial findings, or whether there were clinical findings as seen in Family 1. As we collate more data on ASXL3‐related disorder, we will understand better the natural history of this disorder to allow meaningful conclusions on need for additional screening and ongoing surveillance for features of a connective tissue disorder.

## 4 CONCLUSION

We describe the clinical and molecular characteristics of three families with known familial *ASXL3*‐related disorder. We confirm heritability of this condition but show intrafamilial phenotypic heterogeneity. This report has immediate clinical ramifications with respect to recurrence risk counseling for parents, parental testing and reproductive health advice for patients with *ASXL3*‐related disorder.

## Data Availability

Data sharing is not applicable to this article as no new data were created or analyzed in this study.
